# Arp2/3 Inhibition Induces Amoeboid-Like Protrusions in MCF10A Epithelial Cells by Reduced Cytoskeletal-Membrane Coupling and Focal Adhesion Assembly

**DOI:** 10.1371/journal.pone.0100943

**Published:** 2014-06-26

**Authors:** Yvonne Beckham, Robert J. Vasquez, Jonathan Stricker, Kareem Sayegh, Clement Campillo, Margaret L. Gardel

**Affiliations:** 1 Institute for Biophysical Dynamics, University of Chicago Medical Center, Chicago, Illinois, United States of America; 2 Section of Hematology, Oncology and Stem Cell Transplantation, Department of Pediatrics, University of Chicago Medical Center, Chicago, Illinois, United States of America; 3 James Franck Institute, University of Chicago, Chicago, Illinois, United States of America; 4 Department of Physics, University of Chicago, Chicago, Illinois, United States of America; 5 Laboratoire Physico-Chimie, Institut Curie, Centre de Recherche, Paris, France; 6 Laboratoire Analyse et Modélisation pour la Biologie et l’ Environnement, Université d’Evry Val d’Essonne, Evry, France; Northwestern University Feinberg School of Medicine, United States of America

## Abstract

Here we demonstrate that Arp2/3 regulates a transition between mesenchymal and amoeboid protrusions in MCF10A epithelial cells. Using genetic and pharmacological means, we first show Arp2/3 inhibition impairs directed cell migration. Arp2/3 inhibition results in a dramatically impaired cell adhesion, causing deficient cell attachment and spreading to ECM as well as an 8-fold decrease in nascent adhesion assembly at the leading edge. While Arp2/3 does not play a significant role in myosin-dependent adhesion growth, mature focal adhesions undergo large scale movements against the ECM suggesting reduced coupling to the ECM. Cell edge protrusions occur at similar rates when Arp2/3 is inhibited but their morphology is dramatically altered. Persistent lamellipodia are abrogated and we observe a markedly increased incidence of blebbing and unstable pseuodopods. Micropipette-aspiration assays indicate that Arp2/3-inhibited cells have a weak coupling between the cell cortex and the plasma membrane, and suggest a potential mechanism for increased pseudopod and bleb formation. Pseudopods are not sensitive to reduced in formin or myosin II activity. Collectively, these results indicate that Arp2/3 is not necessary for rapid protrusion of the cell edge but plays a crucial role in assembling focal adhesions required for its stabilization.

## Introduction

Cell migration is an essential physiological process in development, wound healing and immune response. Misregulation of motility can contribute to the progression of inflammatory and vascular diseases as well as cancer metastasis [1], [2]. Migration is a physical process which requires the spatiotemporal coordination of cell protrusion, adhesion and contraction [3]–[5]. It is becoming increasingly clear that diverse modes of cell migration exist which are facilitated by utilization of distinct cytoskeletal machinery [6].

In mesenchymal migration utilized by diverse cell types, including epithelial and fibroblast cells, coordination of protrusion and adhesion at the leading cell edge is facilitated by the lamellipodium. The lamellipodium is a densely branched and treadmilling actin array formed through Arp2/3-mediated actin assembly and cofilin-mediated F-actin severing [5], [7], [8]. Nascent adhesions form within the lamellipodium in an actin-polymerization-dependent manner to couple the actin cytoskeleton to the ECM [3], [9], [10] and facilitate the effective transmission of forces, generated through actin polymerization, to advance the leading edge of the cell [11]. The coordination of adhesion assembly with actin polymerization is not completely understood but is thought to occur via interactions between Arp2/3 with vinculin (DeMali et. al. 2002). Indeed, altered focal adhesion morphology has been observed upon reduction of Arp2/3 activity [12]. In the absence of lamellipodia, alternate actin-polymerization machinery within the lamella [13] or in filopodia [14]–[17] can facilitate cell edge protrusion and adhesion.

Amoeboid-based motility is an alternate migratory phenotype that is only weakly dependent on integrin-mediated adhesion and can occur even in its absence [18]. The forces underlying protrusion in amoeboid migration can originate from actin polymerization at the cell front or actomyosin contraction at the rear [6]. Actomyosin forces decouple the actin cortex from the plasma membrane and generate blebs to drive protrusion of the cell front [19]–[21]. Transitions between mesenchymal and amoeboid migration occur during development [6] and in disease progression [22]. Such transitions require coordination between changes in adhesion assembly, cortex-membrane attachment and cytoskeletal force generation. It was recently shown that these could occur by modulating the protrusive and contractile activity of a carcinoma cell line [23]. However, the underlying changes to cytoskeletal organelles regulating transitions between these disparate modes of migration are less well understood.

Here we demonstrate that a transition between mesenchymal and amoeboid-like protrusion can be induced in MCF10A epithelial cells upon pharmacological inhibition or silencing of Arp2/3. Arp2/3 inhibition abolishes lamellipodial formation and impairs directional migration in MCF10A epithelial cells. Utilizing high resolution live cell imaging, we explore the extent to which this results from changes to protrusive activity or focal adhesion dynamics. We find that the initial stages of cell attachment and spreading in Arp2/3-inhibited cells are impaired by deficient cell adhesion, but not leading edge protrusion. After cell spreading and polarization, Arp2/3 inhibition abrogates nascent adhesion formation near the cell periphery. Focal adhesion growth is not impaired, but mature focal adhesions are poorly coupled to the ECM and undergo large scale motions typically only seen during adhesion assembly or disassembly. Arp2/3 inhibition does not abrogate cell protrusions but the frequency of bleb-like and amoeboid-like pseudopods are significantly increased. The pseudopod protrusions that occur in Arp-inhibited cells require cortical actin, but not myosin II-mediated contractility or high amounts of formin-mediated actin polymerization. Finally, we determine that the strength of cortex-membrane attachment is reduced significantly by Arp2/3 inhibition, suggesting detachment between the plasma membrane and a dynamic, but weakened, actin cortex as a potential mechanism of driving pseudopod protrusion. These experiments demonstrate the important role of Arp2/3 activity in coordinating cell-membrane attachment and adhesion assembly at the cell front to modulate between amoeboid and mesenchymal modes of cell migration.

## Results

### CK-869 disrupts Lamellipodium formation in MCF10A cells

**Figure 1:**
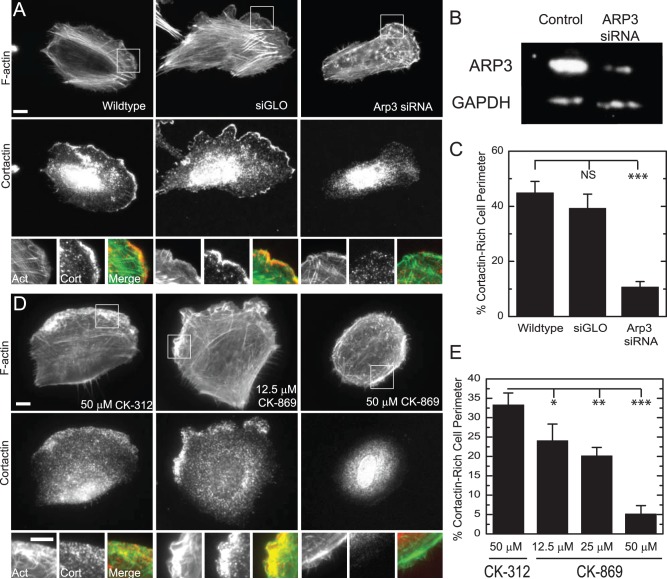
Cortactin-rich laµellipodium is eliminated in MCF10A cells treated with CK-869 or siRNA Arp3. (**A**) Images of actin visualized by fluorescent phalloidin and cortactin immunofluorescence of MCF10A cells, MCF10A cells transfected with siGLO-red fluorescent oligo or with siGLO-red fluorescent and Arp3 siRNA oligos. Scale Bar = 10 µm. (**B**) Western Blot demonstrating that Arp3 siRNA knocked down Arp3 levels to less than 30% of wildtype levels. (**C**) Percentage of cell perimeter containing cortactin staining in MCF10A cells as in A (n = 20, 12 and 35 cells, respectively; error bars = SEM). (**D**) Images of actin visualized by fluorescent phalloidin and cortactin immunofluorescence in MCF10A cells treated with 50 µM of control compound CK-312 or 12.5 and 50 µM of the Arp2/3 inhibitor CK-869 for 4–10 hours. Scale bar = 10 µm. (**E**) Percentage of cell perimeter containing cortactin staining in MCF10A cells as in C (n = 10; error bars = SEM). NS, not significant; *, P<0.05; **, P<0.01; ***, P<0.001 with respect to WT or control.

The lamellipodium is identified by immunofluorescence as a 2–3 µm band of high F-actin density near the cell periphery to which cortactin, an activator of Arp2/3, is localized [12]. Human breast epithelial MCF10A cells plated on fibronectin-coated glass coverslips display a prominent lamellipodium, with approximately 30–40% of the cell periphery staining positive for cortactin (Fig. 1A).

To disrupt lamellipodium formation, we utilized both genetic and pharmacological approaches. We reduced expression of Arp3 to less than 30% of wildtype levels using siRNA (Fig. 1B). These cells showed significantly reduced cortactin staining at the cell periphery compared to the control cells transfected with fluorescently-labeled siGLO (Fig. 1A+C). The pharmacological inhibitors of Arp2/3, CK-869 and CK-666, were also used and directly compared to the control compound CK-312 [24]. MCF10A cells treated with 50 µM CK-312 for >4 hours show a broad cortactin-rich lamellipodium across 30% of the total cell perimeter (Fig. 1D). In MCF 10A cells treated with 12.5 µM CK-869, cortactin-rich lamellipodia still persist, but are typically fragmented and extend only 22% of the cell perimeter. As the CK-869 concentration increases, the percentage of the cortactin-rich cell perimeter decreases to 5% at 50 µM CK-869 (Fig. 1D+E). These data indicate that the pharmacological inhibitor CK-869 is effective in disrupting lamellipodia formation in MCF 10A cells. We saw similar lamellipodial inhibition at extremely high (100 µM) concentrations of another inhibitor, CK-666 (Fig. S1) and when using CK-869 on NIH-3T3 fibroblast cells (Fig. S2).

This reduction of the lamellipodium in MCF10A is dependent on the continued application of CK-869. Cells removed from inhibitor show a return of the lamellipodium after 8 hours in regular media (Fig. S3A).

### ARP2/3 Inhibition Impairs Directed Migration

**Figure 2:**
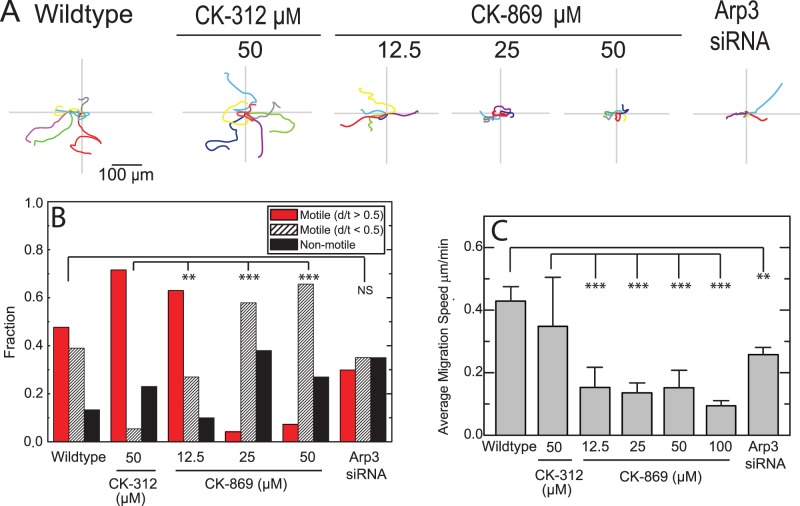
Effect of the Arp 2/3 inhibitor CK-869 and Arp3 knockdown on migration of MCF 10A cells. (**A**) Traces of 6 individual trajectories of MCF10A cells plotted after aligning their starting positions for wildtype cells and cells in 12.5, 25 and 50 µM of CK-869 or siRNA Arp3 cells. Scale bar is 100 µm. (**B**) Fraction of cells that are motile (move greater than 1 cell diameter over 10 hours) or non-motile (move less than 1 cell diameter over 10 hours). Motile cells are segregated into those that have a d/t (d is overall distance traveled; t is total path length) greater or less than 0.5 to identify persistent and non-persistent migration, respectively. (n = 22, 50, 50, 50, 50, 50 and 29 cells respectively).(**C**) Instantaneous migration speed for motile cells in same conditions described in A. (N = 19, 38, 45, 31, 36, 50 and 19 cells respectively). Error bars = SD. NS, not significant; **, P<0.01; ***, P<0.001 with respect to WT or control.

To identify the extent to which Arp2/3 activity is essential for MCF10A cell migration, we performed two-dimensional random migration assays of MCF10A cells plated on fibronectin-coated coverslips for 10 hours (Movie S1). Approximately 75% of wildtype MCF10A cells translocate more than 1 cell diameter over 10 hours and are considered ‘motile’ with an instantaneous velocity of 0.4 µm/min (Fig. 2B). The remaining ∼25% of cells translocate less than 1 cell diameter over 10 hours and are considered “non-motile.” Approximately half of the motile cells exhibit high directional persistence, such that the ratio of the euclidean distance D moved over 1 hr to the total migration distance T is larger than 0.5. (Fig. 2A+B).

Directionally persistent migration is impaired by either treatment with CK-869 or siRNA Arp3. With both genetic (siRNA) and pharmacological (25–50 µM CK-869), the fraction of non-motile cells increases slightly to ∼30–50% (Movie S1, Fig. 2A+B). The instantaneous velocity of the remaining motile cells decreases approximately half to 0.15 µm/min for cells treated with CK-869 and 0.26 µm/min for siRNA Arp3 cells (Fig. 2C). The most dramatic change upon Arp2/3 inhibition is the reduction in the fraction of directionally persistent motile cells to ∼25% for siRNA-treated cells and <5% for 25–50 µM CK-869 (Fig. 2B). Instead, the majority (30–60%) of cells undergo a migration with very poor directional persistence. Thus, Arp2/3 inhibition significantly impairs directional persistence of migration.

### ARP2/3 inhibition affects adhesion during cell spreading, but not the spreading rate

Changes in directional persistence and migration speed could arise from altered cell polarity, adhesion or protrusion. To examine changes in cell adhesion, we incubated cells in suspension with varying concentrations of CK-312 or CK-869. We then plated the cells on tissue culture plastic for 1 hr and determined the relative quantities of cells that remain attached after gentle washing. The relative quantity of attached cells remained constant for cells treated with varying concentrations of the control inhibitor up to 50 µM CK-312 and up to 25 µM CK-869. Surprisingly, we found a 50% reduction in attached cells for those treated with 50 µM CK-869, indicating a role of Arp2/3 in the initial stages of cell adhesion to ECM.

**Figure 3:**
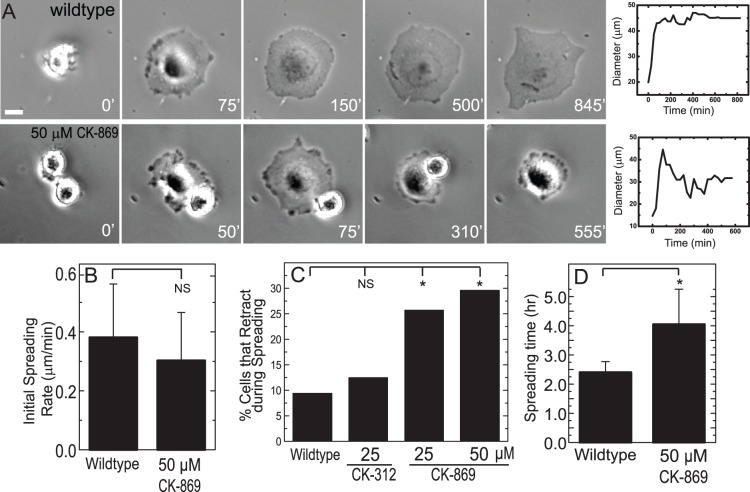
Arp2/3 inhibited cells show defects in spreading. (**A**) Time series of untreated (wild type) cells or cells treated with 50 µM of CK-869; time indicated in minutes. Panels to the right show representative graph of spread area versus time for a cell in the cells presented in A. Scale bar = 10 µm (**B**) Rates of cell spreading in wildtype MCF10A cells or cells treated with 50 µM CK-869. Rates of spreading were calculated from the slopes of kymographs of the cell edge (n = 22 cells for wild type and 21 cells for CK-869). (**C**) Fraction of cells that retracted after reaching their maximum spread area. The ratio of the full spread area (FSA) to the smallest spread area (SSA) was calculated and if FSA/SSA >2, the cell was included as a retraction (n = 53 untreated cells, n = 64 for 50 µM control compound (CK-312) and n = 35 and 27 cells for 25 and 50 µM CK-869, respectively). (**D**) Bar graph of the time in hours to reach maximum spread area for untreated MCF10A cells or cells treated with 50 µM CK-869. NS, not significant; *, P<0.05 with respect to WT or control.

To examine the impaired cell adhesion more closely, we analyzed phase contrast images of cells attaching and spreading on fibronectin-coated glass coverslips (Movie S2, Fig. 3A). After attachment, untreated MCF10A cells uniformly spread at a rate of 0.4 µm/min over a course of ∼2 hrs (Fig. 3A+B), after which time they polarize and begin to migrate (data not shown). In 10% of cells, spreading is interrupted by retraction and cell rounding (Fig. 3C). By contrast, cell spreading is interrupted by detachment and rounding in 25–30% of cells treated with 25 µM CK-869 (Fig. 3A–C, Movie S2). Unexpectedly, the rate of spreading remains unchanged upon CK-869 treatment (Fig. 3B). While the initial spreading rate is not reduced by Arp2/3 inhibition, the intermittent retraction and rounding result in a much longer time, 4 hrs, to achieve steady state spread area (Fig. 3D). Thus, consistent with our earlier observations, Arp2/3 inhibition results in impaired initial cell-ECM attachment and adhesion but does not impact the rate of protrusion significantly.

### ARP2/3 Inhibition Impairs Adhesion Assembly and Coupling to ECM

**Figure 4:**
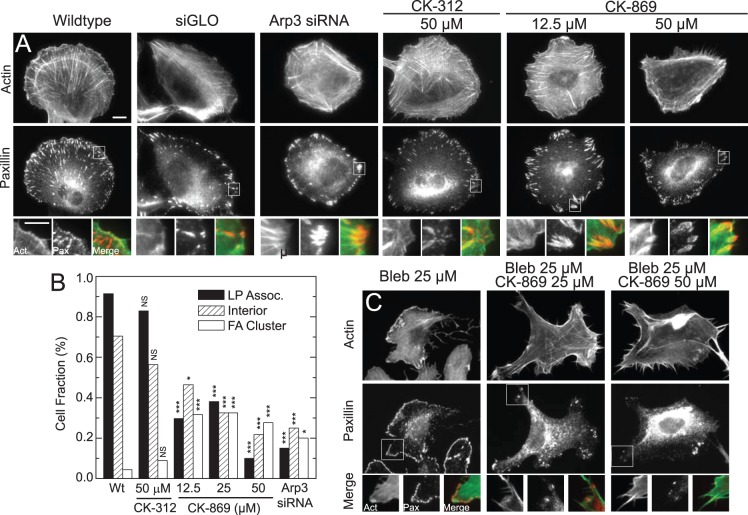
Arp2/3 inhibited cells show fewer nascent focal adhesions, fewer interior focal adhesions, and large focal adhesion clusters. (**A**)Top panel consists of images of F-actin visualized by fluorescent phalloidin (top row) and paxillin immunofluorescence (middle row) and enlargements of regions of interest (bottom row) for wild type, siGlo cells, siRNA Arp3 and cells treated for four hours with inidicated concentrations of either control (CK-312) or Arp2/3 inhibitor (CK-869). (**B**) Plot of focal adhesion phenotypes as a function of inhibitor concentration or siRNA. Cells with lamellipodia-associated adhesions were cells with focal adhesions less than 0.3 µm^2^ found in lamellipodia. Cells with interior adhesions were cells with focal adhesions found at least 5 µm away from the cell edge. FA cluster are cells containing dense clusters of focal adhesions, as shown in inset of siArp3 and 12.5 µM CK-869 images. All focal adhesions in the cells were counted (n = 43, 32, 45, 38, 48, 19) NS, not significant; *, P<0.05; ***, P<0.001 with respect to WT or control. (**C**) Images stained as in (A) treated with 25 µM Blebbistatin and the indicated concentration of CK-869.

We hypothesized that the spreading defects observed upon Arp2/3 inhibition resulted from impaired focal adhesion assembly. To examine the distribution and morphology of adhesions, we examined images of fixed cells stained for paxillin, a focal adhesion protein that is abundant at all stages of a focal adhesion’s life cycle, and F-actin. In control MCF10A cells, the lamellipodium co-localizes with paxillin staining of small, sub-micron sized adhesions (Fig. 4 A) in 90% of cells (Fig. 4B). Proximal to the lamellipodium, paxillin-rich adhesions elongate into plaques that are several microns in length and approximately 1 µm in width. In 70% of cells, adhesions are also observed throughout the cell interior under the nucleus (Fig. 4A+B). The frequency and morphology of adhesions in the lamellipodia and cell interior remains unchanged in cells treated with 50 µM CK-312 (Fig. 4A+B).

Focal adhesions still form in cells treated with 12.5 µM CK-869, but their distribution and morphology is dramatically changed. In this condition, only 30% of cells display sub-micron adhesions at the cell periphery (Fig. 4A+B) that would normally be present in a lamellipodium. Moreover, the fraction of cells with adhesions in the cell interior is also dramatically reduced (Fig. 4A+B). Elongated focal adhesions at the lamella periphery still exist but their morphology is altered. While their length remains unaffected, focal adhesion plaques tend to form dense clusters which can make individual plaques that are several micro-meters in width (Fig. 4A+B). The frequency of these phenotypes is enhanced at higher concentrations of CK-869 or siRNA Arp3 (Fig. 4A+B). Thus, even incomplete inhibition of Arp2/3 with low concentrations of CK-869 has a dramatic adhesion phenotype.

The reduced number of sub-micron adhesions at the cell periphery suggests that Arp2/3 inhibition impairs nascent adhesion assembly. Nascent adhesions are myosin-independent adhesions that assemble at the leading edge, and thus, are insensitive to inhibition of myosin II activity [9]. In control cells, inhibiting myosin II ATPase activity with the pharmacological inhibitor Blebbistatin abolishes both actin bundles within the lamella and elongated focal adhesions. However, a dense band of nascent adhesions remain at the lamellipodial base [9], [11] (Fig. 4C). By contrast, in cells treated with both 25 µM blebbistatin and CK-869, these adhesions are abolished (Fig. 4C).

**Figure 5:**
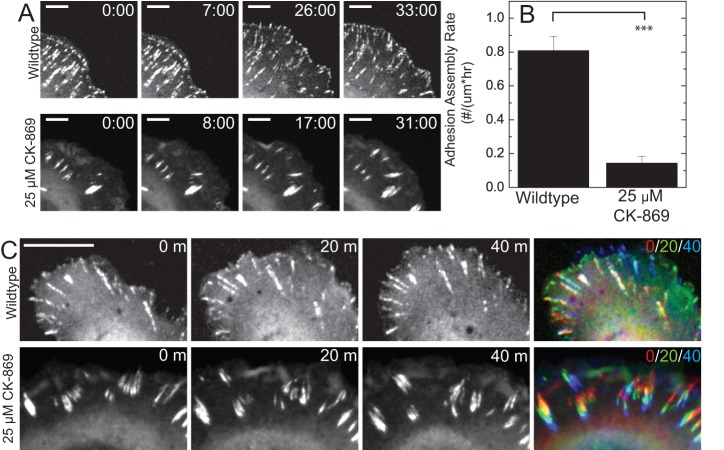
Arp2/3 inhibition impairs nascent adhesion assembly. (**A**) Time-lapse images of paxillin visualized by expression of GFP-paxillin in normal cells and cells treated with 25 µM CK-869. Scale bar is 5 µm. Time indicated is min:sec (**B**) Plot of the adhesion assembly rate, measured as the number of new focal adhesions formed normalized by length of cell edge and total time of formation (n = 6 and 3 cells; error bars = SEM). ***, P<0.001 with respect to WT. (**C**) Time-lapse images of GFP-paxillin visualized by expression of GFP-paxillin in wildtype and 25 µM CK-869 cells. The color combined image represents the time series as a color progression from red to green to blue. Scale bar is 10 µm.

To directly measure changes in adhesion assembly dynamics, we examined adhesion assembly at the leading edge of U2OS cells expressing GFP-Paxillin using time-lapse confocal imaging. In control cells, nascent adhesions assemble at the cell periphery at a rate of 0.8 adhesions per micro-meter of cell periphery per second (Fig. 5A+B, Movie S3). By contrast, cells treated with 25 µM CK-869 also show ruffling of the cell periphery but ∼0.1 adhesion/(µm•s) nascent adhesions assemble (Fig. 5A+B, Movie S3). Thus, Arp2/3-mediated actin polymerization is crucial to nascent adhesion assembly.

Elongated focal adhesion plaques are typically immobile with respect to the ECM to maintain a firm connection to the ECM. In control cells, we find that the distal tip of focal adhesions within the lamella remains unchanged over the course of 30 min (Fig. 5C). By contrast, the distal tip of adhesions in cells treated with 25 µM CK-869 move proximally several microns over the course of 30 min, tending to rearrange the location and direction of adhesion plaques. The adhesion plaques themselves remain stable over the course of 40 min, but their direction and location within the cell changes. Without further analysis, it is impossible to ascertain whether the adhesion plaques are actually undergoing translocation or treadmilling. However, Arp2/3 inhibition reduces the coupling of adhesion plaques to the ECM to result in gross FA movements only typically seen during adhesion assembly [25] and disassembly [26].

### ARP2/3 Inhibition Induces the Formation of Unstable, Rapidly protruding Pseudopods

**Figure 6:**
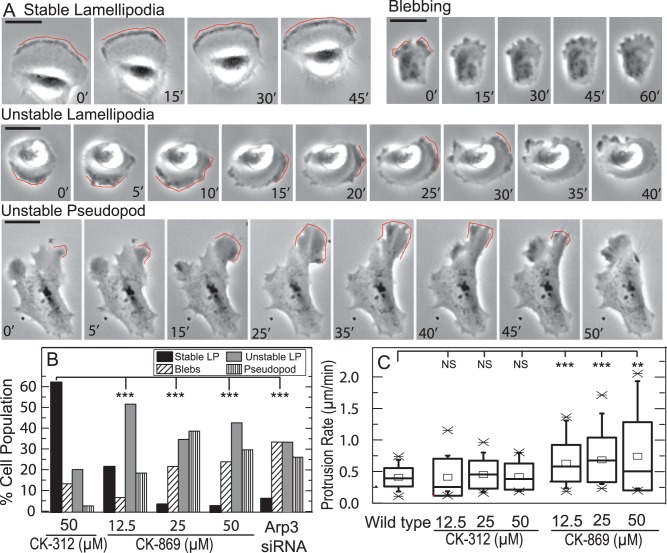
Protrusions observed in MCF10A cells at different concentrations of the Arp2/3 inhibitor, CK-869. (**A**) Phase contrast images of the protrusion phenotypes observed. A broad lamellipodia that remains stable for ∼1 hr drives persistent migration in wild type cells (Stable LP). Small (1 µm) and dynamic blebs drive local protrusions in cell treated with 50 µM CK-869. Unstable, lamellipodia-like protrusions (Unstable LP) drive local protrusion in cell treated with 50 µM CK-869 over ∼10 min time periods. Protrusions are unstable and appear as a travelling wave along the cell periphery. Large pseudopodial protrusions rapidly extend ∼30 µm over ∼30 min but are unstable and retract shortly thereafter. Red line indicates protrusion of interest for each phenotype. Scale bar is 25 µm. (**B**) Percentage of cells exhibiting the protrusion phenotype described in A for untreated cells, cells treated with 12.5, 25 and 50 µM of CK-869, 50 µM CK-312 or cells transfected with Arp3 siRNA n = 39, 30, 24, 21 and 27 cells respectively, (**C**) Box plot of protrusion rate for untreated cells, cells treated with 12.5, 25 and 50 µM of CK 312, and cells treated with 12.5, 25 and 50 µM of CK-869 and imaged for 16 hours. Square  =  mean, X = 1% and 99% quantile. Box is median and SD. n = 39, 13, 14, 17, 30, 24 and 21 cells respectively; NS, not significant; **, P<0.01; ***, P<0.001 with respect to WT or control.

We were surprised to observe that Arp2/3 inhibition did not impact the rate of cell spreading (Fig. 3B), as the forces driving protrusion in early spreading are typically thought to originate in the lamellipodia. Thus, we wished to explore the nature of cell protrusions of migrating cells more closely (Movie S4). In wildtype MCF10-A cells, 60% of cells display a persistent and stable lamellipodium, which is identified in phase contrast images as a dark band co-localized with the leading cell edge (Fig. 6A+B, Movie S4). The rate of lamellipodial protrusion matches exactly that of the cell migration speed (0.4 µm/min) (Fig. 6C), indicating a tight coupling between protrusion and migration speed observed in highly motile cell types [27]. Other protrusion phenotypes are observed in wild type cells, but are much less common. Approximately 20% of cells display an “unstable lamellipodia” (Movie S4), characterized by a phase-dense band that does not lead to a persistent protrusion but rather translocates as a “wave” along the cell perimeter (Fig. 6A+B). In 10% of wildtype cells, especially those that are poorly spread, bleb-like protrusions (Movie S4) are observed and are identified as short-lived (<2 min), phase-dense protrusions that are less than 1 µm in size (Fig. 6A+B). In the absence of further experiments, we cannot definitely state the extent to which these bleb-like protrusions lack any sort of cortical actin, as is the case with canonical blebs.

Upon Arp2/3 inhibition, the incidence of a “stable lamellipodia” phenotype is reduced to <20% for 12 µM CK-869 and 2–5% for 25–50 µM CK-869 or siRNA Arp3 cells and the “unstable lamellipodia” and “blebbing” protrusions are more prominent (Fig. 6B, Movie S1). In cells treated with both 50 µM CK-869 and Arp3 siRNA the additive effects of the two methods of inhibition result in no cells containing a “stable lamellipodium” (Fig. S4). The most dramatic change was the appearance of a new protrusion phenotype we term “unstable pseudopod” that is only rarely (2% of population) observed in wildtype MCF10A cells. These protrusions were initiated in a small region of the cell periphery and then appeared to rapidly “inflate” and drive a fast extension of the cell periphery over ∼30 min at rates up to 1 µm/min (Fig. 6A, Movie S4). After a period of extension, the protrusion retracted rapidly and, in some instances, appeared correlated to the initiation of another protrusion in a distal region of the cell. These rapid protrusions are relatively short-lived, typically under 30 minutes. The percentage of cells exhibiting large, unstable pseudopodial protrusions increased to >30% of cells treated with 25 µM CK-869 or siRNA Arp3 (Fig. 6B). The appearance of this novel protrusion phenotype increased the average protrusion rate (and its variability) in cells treated with CK-869, as compared to wild type cells or the control compound CK-312 (Fig. 6C, Movie S1). Similar protrusion phenotypes were observed with high concentrations of CK-666 (Fig. S1). Thus, Arp2/3 inhibition results in enhanced, but more variable, cell edge protrusion. However, because the pseudopods are poorly adherent, they are not efficient modes of cell edge advance.

### Pseudopodial protrusions in Arp2/3-inhibited cells are independent of myosin II and high levels of formin activity, but require an intact actin cortex

**Figure 7:**
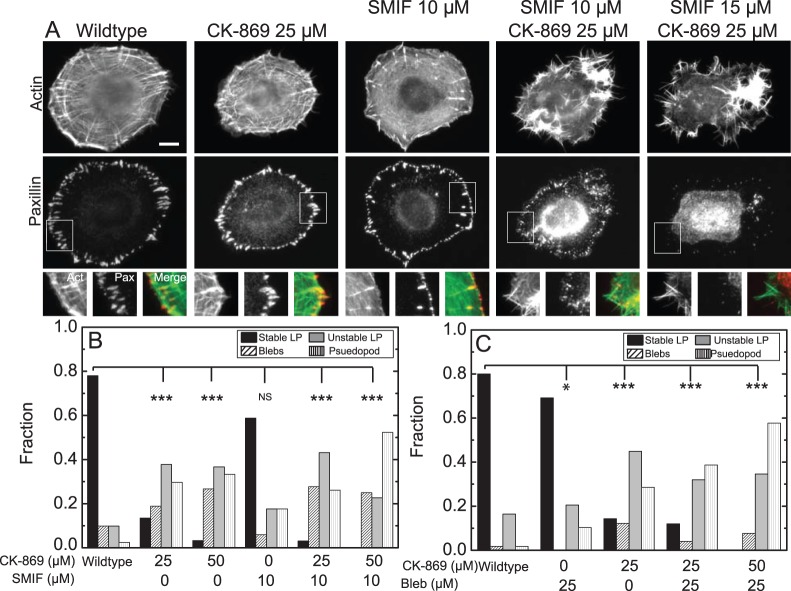
Myosin or Formin Inhibition does not abrogate pseudopodial protrusions in Arp2/3 inhibited cells. (**A**) Images of F-actin visualized by fluorescent phalloidin (top row) and paxillin immunofluorescence (middle row) and enlargements of regions of interest (bottom row) indicated by square for wildtype MCF10A cells or cells treated with CK-869 and the formin inhibitor SMIFH2 at the indicated concentrations. (**B**) Fraction of MCF10A cells treated with indicated concentrations of CK-869 and SMIFH2 displaying the protrusion phenotypes as in Figure 6 (n = 41, 37, 30, 17, 65 and 44 cells respectively). Cells were treated for four hours prior to imaging. (**C**) Fraction of MCF10A cells treated with indicated concentrations of CK-869 and myosin ATPase inhibitor blebbistatin displaying the protrusion phenotypes as in Figure 6 (n = 55, 39, 49, 75, 26 cells respectively). Cells were treated with CK-869 for four hours prior to imaging and were treated with blebbistatin at the start of imaging. NS, not significant; *, P<0.05; ***, P<0.001 with respect to WT or control.

To elucidate the mechanisms regulating pseudopodial protrusions, we decided to explore whether formin or myosin II activity contributed to their formation. Treatment of MCF10A cells with the 10 µM formin inhibitor SMIFH2 [28] did not impact lamellipodia formation or nascent adhesion formation at the cell edge but did impact stress fiber assembly and focal adhesion maturation (Fig. 7A), consistent with previous results [29]. The protrusion phenotypes observed in formin-inhibited cells were largely similar to wild type cells, although a slightly higher incidence of pseudopods were observed (Fig. 7B, Movie S5). Simultaneous inhibition of Arp2/3 and formin activity dramatically altered cell edge morphology, increasing the incidence of filopodial-like bundles at the periphery and abrogating adhesion formation (Fig. 7A). The incidence of pseudopodial or blebbing protrusions was still high in these cells (Fig. 7B, Movie S5), suggesting that formin-mediated actin polymerization is not playing a crucial role. However, when cells are treated with higher doses of SMIFH2 and CK-869 simultaneously, cells round up and protrusions stop.

To determine whether pseudopods require myosin II ATPase activity, we treated cells with saturating doses of blebbistatin and CK-869. Protrusion phenotypes were also observed in cells treated with both CK-869 and blebbistatin, indicating that pseuopod formation is not sensitive to myosin II ATPase activity (Fig. 7C, Movie S5).

### Membrane detachment and blebbing occurs at low stresses in Arp2/3 inhibited cells

**Figure 8:**
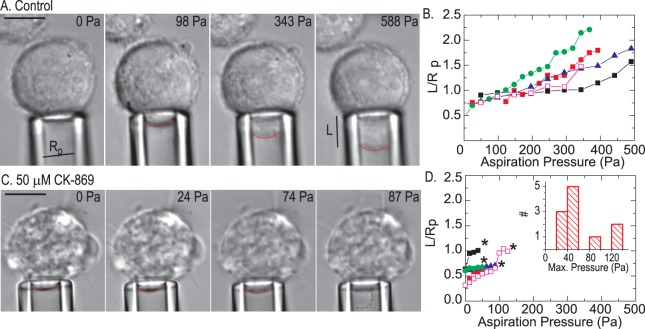
Arp 2/3 inhibition induces bleb formation at low external pressures. (**A**) Phase contrast images of wildtype cells in a micro-pipette aspiration experiment as the aspiration pressure is increased from 0 Pa to 588 Pa. Aspiration pressure indicated in upper right. Scale bar is 3 µm. Cell contour within the micropipette indicated by red line. The pipette radius R and the length L of cell extended into the pipette are indicated in the images. (**B**) L/Rp as a function of aspiration pressure for wildtype cells; each data series reflects data (**C**) Phase contrast images of cells treated with the Arp inhibitor CK-869 in an aspiration experiment with cell contour within the pipette indicated. In the highest pressure (87 Pa), a bleb abruptly forms within the pipette, indicated by dashed red line. Scale bar is 3 µm. (**D**) L/Rp as a function of aspiration pressure for Arp inhibited cells. Note that bleb formation occurs at low pressures (stars), with a mean of 45 Pa. Inset, histogram of pressures at which blebs form.

The membrane protrusions we see with Arp2/3 inhibition include an increase is cell blebbing, which arises from reduced attachment between the plasma membrane and cortex [18]. We speculated that pseudopods may arise from a similar mechanism. To probe the strength of cortex-membrane interactions and promote the formation of artificially induced blebs, we used micro-pipette aspiration to apply external pressure to non-adhered cells [30]–[32]. As pressure applied to the cell cortex increases from 0 to 98 Pa, the wild type (untreated) cell starts to deform as it is pulled into the pipette (Fig. 8). The extent of deformation increases as the applied pressure increases up to 500 Pa. By contrast, in cells treated with 50 µM CK-869, induction of a bleb occurs at very low aspiration pressures in place of cortical deformation. The average stress required to induce blebbing is 45 Pa (Fig. 8). Such pressure-induced bleb formation is not observed in control cells or in cells treated with SMIFH2 (Fig. S5), indicating that reducing cortical actin density by formin inhibition does not weaken cortex-membrane attachment sufficiently to induce bleb formation. Thus, Arp2/3 plays an important role in coupling the actin cortex to the plasma membrane.

## Discussion

Recent studies have demonstrated numerous mechanisms by which cells compensate for impaired lamellipodia by utilizing alternate cytoskeletal machinery. In fibroblasts, inhibition of Arp2/3 induces migration via a filopodial-based mechanism [16], [17]. In a carcinoma cell line, Arp2/3 inhibition induces the transition between a lamellipodial and bleb-based migration driven by actomyosin contractility [23]. In the normal breast epithelial cells used in this current study, we do observe an increase in the incidence of bleb-like protrusions, but we also observe the increase of a weakly adherent pseudopod that is independent of myosin activity. Filopodial-based migration does not appear to play a predominant role in driving protrusion in our epithelial cells. These results demonstrate the diversity of cytoskeletal modules that can compensate for impaired lamellipodial activity to drive cell protrusion. However, the predominant module that appears upon Arp2/3 inhibition is highly cell type dependent.

Our studies also provide further insight into the role of the lamellipodium in nascent adhesion assembly. Previous work has shown reduced adhesion density and function upon inhibition of Arp2/3 [12], [16], but underlying mechanisms were not completely understood. In current models for nascent adhesion nucleation, two scenarios have been proposed [4]. In the first model, integrin activation and binding to the ECM initiates a cascade of signaling for further recruitment and clustering. In the second model actin polymerization provides the template for formation of adhesion complexes, with integrin activation and binding to the ECM occurring later. Our work is consistent with a previous study [9] supporting the later model of actin polymerization-dependent adhesion assembly. Here we show that nascent adhesion assembly specifically requires Arp2/3, as inhibition of formin activity has no impact on nascent adhesion formation. This adhesion defect is observed even at low doses of the Arp2/3 inhibitor which are insufficient to fully abrogate lamellipodial activity. Our data also shows that Arp2/3 activity is necessary for initial cell attachment, even before a canonical lamellipodia is set up. Future work will need to identify the molecular mechanisms by which Arp2/3 regulates nascent adhesion assembly, although interactions with the focal adhesion protein vinculin have been proposed to play an important role [33], [34].

We also identify a potentially important role for Arp2/3 in mature focal adhesions. While focal adhesion elongation (and presumed maturation) is not affected by Arp2/3 inhibition, the stability of mature adhesions against the ECM is impaired, resulting in either a translocation or treadmilling of mature adhesions. Such FA translocation is typically reserved for adhesions at the initial stages of assembly or during adhesion disassembly [25], [26]. This indicates that Arp2/3 may continue to play a role in stabilizing integrin-ECM attachment in mature focal adhesions and may be the cause of deficient adhesion reorganization recently reported [16]. A possibility we find intriguing is that regulation of actin polymerization dynamics at focal adhesions may also be a control parameter utilized to regulate adhesion disassembly at the trailing edge of cells. This is consistent with the emerging role of actin architecture and polymerization dynamics as a regulator of several stages of adhesion assembly and maturation.

Our data suggest that the mechanism by which a pseudopod and bleb protrusion phenotype arises from Arp2/3 inhibition is by reduced membrane-cortex attachment. In contrast to recent work [23], these phenotypes are not myosin-dependent. These differing results may reflect an overall difference in the level of myosin contractility in different cell types. We speculate that Arp2/3 plays a prominent role in maintaining cortex-membrane attachment and, in its absence, holes develop in the cortex that, due to osmotic pressure in the cell, drive membrane protrusions. This is consistent with recent studies showing that cells depleted of Arp2/3 are hypersensitive to osmotic stress [35]. Decreasing the cortical attachment to the membrane previously has been shown to affect the variety and prevalence of certain types of cell protrusions (Diz-Munoz et al, 2010) and we speculate that Arp2/3 plays an important role in actin cytoskeletal remodeling events to maintain efficient coupling to the plasma membrane [36].

Finally, our results also call into question the extent to which lamellipodial-generated forces regulate the rate of cell protrusion, especially in early cell spreading. We did not find that the rate of advance diminished with reduced Arp2/3 activity, consistent with previous findings in fibroblasts [17]. This result is consistent with several studies that show that cell spreading or lamellipodial advance is regulated by membrane tension [37] and suggests a model whereby the lamellipodia serves to “fill the gap” in regions of weak membrane-cortex attachment or low membrane tension. This indicates the possibility that maintenance of the leading cell edge has less to do with local Arp2/3 activation but spatial regulation of membrane tension.

It is becoming increasing clear that transitions between different migratory phenotypes can be regulated by both environmental and cytoskeletal cues. Here we’ve identified a central role of Arp2/3 both in adhesion assembly and coupling the cortex to the ECM. Although we have interpreted these effects largely independently, it is clear that interdependence between adhesion assembly, cortical tension and cortical actin dynamics impact leading edge morphology. It will be interesting to see if local modulation of Arp2/3 activity is utilized to modulate migratory phenotypes during development or disease states.

## Materials and Methods

### Cell Culture and Reagents

MCF10A human breast epithelial cells (ATCC) were cultured in DMEM/F12 50∶50 (Mediatech) supplemented with 10% fetal bovine serum (HyClone), penicillin-streptomycin (Gibco), 20 ng/ml EGF (BD BioCoat), 0.5 µg/ml hydrocortisone (MP Biomedicals), 10 µg/ml insulin (Roche). U2OS human osteosarcoma cells (ATCC) were cultured in McCoy’s 5A medium (Sigma) supplemented with 10% fetal bovine serum (HyClone), penicillin-streptomycin (Gibco), and 2 mM L-glutamine (Gibco). MCF10A cells were transfected with plasmid DNA constructs encoding for GFP-actin (gift of the Gary Borisy Lab, Northwestern University), mApple-paxillin (gift of the Mike Davidson Lab, Florida State University), and EGFP-paxillin [38] using the transfection reagent FuGENE 6 (Roche). U2OS cells were transfected with EGFP-paxillin using the FuGENE 6 protocol. The following antibodies were used: mouse anti-paxillin (Millipore); Cy5 donkey anti-mouse and Cy2 donkey anti-mouse (Jackson ImmunoResearch), rabbit anti-cortactin (Cell Signaling); mouse anti-FAK (BD Transduction Laboratories). The ARP2/3 inhibitors CK-869 and CK-666 and control compounds CK-312 and CK-689 were purchased from Calbiochem. Blebbistatin (Sigma, St. Louis, MO) treated cells were treated at 25 µM for 30 minutes before fixation or at the start of live imaging. Cells treated with the formin inhibitor SMIFH2 (a gift from D. Kovar, University of Chicago, Chicago, IL; [28] were treated for four hours prior to fixation or live imaging at 10–15 µM.

### siRNA Knockdown

ON-TARGETplus SMARTpool siRNA constructs for human ARP3 and siGLO transfection indicator (Dharmacon) were transfected as per manufacturer’s protocol. Knockdown cells selected for analysis had at least four times the background levels of siGLO red.

### Immunofluorescence

Cells were plated on glass coverslips coated with 5 µg/ml human plasma fibronectin (Millipore) for 24 hours prior to fixation. For immunofluorescence experiments with the control compound and the inhibitor, cells were placed in media containing the different concentrations of inhibitors or control compounds and incubated for several hours or overnight. Coverslips were rinsed in cytoskeleton buffer (CB; 10 mM MES, 3 mM MgCl2, 1.38 M KCl, and 20 mM EGTA) and then fixed, blocked and permeablized in a 4% paraformaldehyde (Electon Microscopy Science), 1.5% BSA (Fisher Scientific) and 0.5% Triton X-100 in CB at 37°C for 10 min. Coverslips were washed 3x in PBS and incubated with primary antibody (1∶100) in a blocking solution (1.5% BSA and 0.5% Triton-X100 in CB) for one hr at room temperature. After the primary antibody incubation, coverslips were washed 3x in PBS and incubated with an appropriate fluorescent secondary antibody (1∶1000) and phalloidin (1∶1000) in blocking solution for at least 1 hr at room temperature. After the secondary antibody incubation coverslips were washed in PBS and mounted on glass slides. Coverslips were mounted in ProLong Gold (Invitrogen) and sealed with nail polish.

### Live Cell Imaging

Coverslips were incubated with 5 µg/mL human plasma fibronectin (Millipore) for one hour before cell plating. For migrations studies of varying fibronectin concentrations, coverslips were then incubated with a 1% BSA solution. Cells were plated for four hours prior to imaging. Cells were imaged for 16 hours in media supplemented with 10 mM HEPES.

### Microscopy

Live cell imaging was performed on a temperature-controlled inverted Nikon Ti-E microscope with a Lumen 200 Pro light source (Prior) and an HQ2 cooled CCD camera (Roper Scientific) controlled via Metamorph acquisition software (MDS Analytical Technologies). Live cell movies of transfected cells were collected on an inverted Nikon Ti-E microscope with a CSU-X confocal scanhead (Yokogawa), laser merge module containing a 491 laser line (Spectral Applied Research) and an HQ2 cooled CCD camera (Roper Scientific). Motility and protrusion data was obtained using either a 10X or 20x objective (Nikon); Immunofluorescence Images were obtained using a 40x 1.3 NA Plan Fluor objective (Nikon).

### Adhesion and Spreading Assays

For adhesion assays, cells were treated in suspension with different concentrations of the compounds, allowed to adhere to the surface of a tissue culture dish for one hour, washed and the number of adherent cells counted and presented as a fraction of untreated cells (10X lens, 10 fields/condition). For cell spreading assays, cells were treated with CK-312 or CK-869 in suspension, plated on coverslips coated with 5 µg/ml fibronectin for imaging.

### Analysis

Percentage cortactin-rich cell perimeter was calculated by measuring the areas of the cell perimeter that contained cortactin staining and dividing it by the entire cell perimeter. These measurements were done with Image J software.

Focal adhesion phenotypes were assessed by counting all focal adhesions present in fixed and stained cells for the focal adhesion traits described in the figures. Focal adhesion assembly was computed by counting all new focal adhesions in a region of protruding cell and dividing by the protrusion size and time interval.

For motility assays, we defined motile cells as cells that move by more than one cell diameter during the 16 hrs of imaging. The *x*, *y* positions of individual cells obtained by tracking in Metamorph and were used to calculate distance traveled in each step {*d* = √[(*x_t_*_+τ_–*x_t_*)^2^+(*y_t_*_+τ_–*y_t_*)^2^]} and linear distance from starting point to end point {*D* = √[(*x_end_*–*x_start_*)^2^+(*y_end_*–*y_start_*)^2^]} The sum of *d* for all steps (*T*) gives total distance traveled, and *D*/*T* ratio provides a quantitative measure of directional persistence; this measurement is calculated over a 4 hour time interval. Step distance *d* was used to calculate migration velocity during each step (*v*). Average migration speeds and standard deviations for each condition were calculated (N = ∼50 cells for each condition),

The data for the cell spreading assays was analyzed using MetaMorph software. Rates of spreading were calculated from the slopes of kymographs of the cell edge (n = 22 for control and 21 for CK-869). The ratio of the full spread area (FSA) to the smallest spread area (SSA) was calculated and if FSA/SSA>2, the cell was included as a retraction (n = 53 untreated cells, n = 64 for 50 µM control compound and n = 35 and 27 cells for 25 and 50 µM CK-869, respectively).

### Statistical analysis

To assess statistical significance (Figures 1C, E; 2C; 3B, D; 5B; and 6C), we used independent two-sample Student’s *t* tests of the mean to determine the significance with respect to WT or Control. To assess statistical significance between observed phenotypes in distinct populations (Figures 2B; 3C; 4B, 6B; and 7B, C), we used Chi squared analysis to show that cell fraction distribution was significantly different from an the control population. The resulting P-values are indicated by *, P<0.05; **, P<0.01; ***, P<0.001 with respect to WT or control.

### Micropipette Aspiration

Micropipette aspirations were visualized on an inverted Nikon Ti-E microscope with with a Lumen 200 Pro light source (Prior) and an HQ2 cooled CCD camera (Roper Scientific). The observation chamber is made of two glass coverslips separated by a Parafilm spacer. A glass micropipette, pulled from borosilicate capillaries (0.7/1.0 mm inner/outer diameter, Kimble, Vineland, NJ) with a laser puller (Sutter P2000) and microforged (DMF1000, World Precision Instruments, Aston, UK) to a diameter of 4–6 µm, is inserted into the chamber. The chamber is filled with a 0.5 mg/mL casein solution for 30 minutes to passivate all glass surfaces. Then the casein solution is replaced by cell culture medium. The aspiration pressure is adjusted by controlling the height of a mobile water tank connected to the micropipette. A cell is selected and the micropipette is brought close to the cell membrane with a 0 aspiration pressure. Then the aspiration pressure is increased from 0 Pa to 588 Pa while images of the cell are recorded. The length of the cell portion aspirated inside the micropipette is then measured with ImageJ.

## Supporting Information

Figure S1 **Arp2/3 inhibition with alternate inhibitor CK-666.** (**A**) Images of actin visualized by fluorescent phalloidin and cortactin and paxillin immunofluorescence in MCF10A cells treated with 0, 50, 100 or 200 µM of the Arp2/3 inhibitor CK-666. (**B**) Fraction of MCF10A cells treated with 0, 50, 100 or 200 µM of the Arp2/3 inhibitor CK-666 that display the protrusion phenotypes as defined in Figure 6 (n = 93, 45,107 and 86 cells respectively). NS, not significant; ***, P<0.001 with respect to WT.
(EPS)

Figure S2 **Arp2/3 inhibition of NIH 3T3 fibroblasts with inhibitor CK869 decreases cortactin staining in the lamellipodium.** (**A**) Images of actin visualized by fluorescent phalloidin and cortactin and paxillin immunofluorescence in NIH 3T3 cells treated with 25 or 50 µM of the Arp2/3 inhibitor CK-869. Scale bar is 20 µm. (**B**) Percentage of cell perimeter containing cortactin staining in NIH 3T3 cells as in A (n = 10,10 and 9 respectively; error bars = SEM). **, P<0.01; ***, P<0.001 with respect to control.
(EPS)

Figure S3 **Washout of CK-869 demonstrating reversibility of inhibition.** (**A**) Images of actin visualized by fluorescent phalloidin and cortactin and paxillin immunofluorescence in MCF10A cells that were treated with 50 µM of CK-312 or 50 µM of CK-869. CK-869 cells were then incubated in fresh media without CK-869 for four or eight hours. Some of the CK-869 treated cells were incubated in media containing 20 µM latrunculin for 30 minutes before washout. Scale bar is 20 µm. (**B**) Percentage of cells exhibiting the protrusion phenotype described in Figure 6A for MCF10A cells that were treated with 50 µM of CK-312 or 50 µM of CK-869. Washout cells were then incubated in fresh media without CK-869 for four hours (n = 64, 52, and 47 respectively). NS, not significant; ***, P<0.001 with respect to control.
(EPS)

Figure S4 **Arp2/3 inhibition and Arp3 knockdown using siRNA display the same protrusion phenotypes with an additive effect.** Fraction of MCF10A cells displaying the protrusion phenotypes as defined in Figure 6 after treatment with indicated concentrations of CK-869 and transfected with siGLO or siGLO and Arp3 siRNA oligos (51, 37, 66, 67, 31 cells respectively). *, P<0.05; **, P,0.01; ***, P<0.001 with respect to control, CK-869 only or siRNA only as shown.
(EPS)

Figure S5 **Formin inhibition does not induce bleb formation at low pressures.** L/Rp as a function of aspiration pressure for cells treated with 15 µM of SMIFH2, calculated as in Figure 8.
(EPS)

Movie S1 **Arp2/3 inhibited cells show migration and protrusion defects.** Wildtype MCF10A cells and cells treated with 50 µM of CK-312, 12.5 or 50 µM of CK-869 or transfected with ARP 3 siRNA oligo. Scale bar = 20 µm. Time is indicated in hr:min.
(MOV)

Movie S2 **Arp2/3 inhibited cells show defects in spreading.** Wildtype MCF10A cell and cells treated with 50 µM of CK-869 during spreading. Scale bar = 100 µm. Time is indicated in hr:min.
(MOV)

Movie S3 **Arp2/3 inhibited cells form fewer nascent focal adhesions than control cells.** U2OS cells transfected with paxillin and either untreated or treated with 25 µM CK-869 for four hours before imaging. Scale bar = 20 µm. Time is indicated in min:sec.
(MOV)

Movie S4 **Protrusion phenotypes seen in Arp2/3 inhibited cells.** MCF10A cell treated with 50 µM of CK-312 showing stable lamellipodium. MCF10A cells treated with 50 µM of CK-869 showing unstable lamellipodium, blebbing and unstable pseudopod. Scale bar = 20 µm. Time is indicated in min:sec.
(MOV)

Movie S5 **Protrusions seen in Arp2/3 inhibited cells are not formin or myosin dependent. MCF10A cells treated with 10 µM SMIFH2 or with 10 µM SMIFH2 and 25 µM of CK-869.** MCF10A cells treated with 25 µM of blebbistatin or with 25 µM of blebbistatin and 25 µM of CK-869. Scale bar = 40 µm. Time is indicated in hr:min.
(MOV)
